# Demographic and Clinical Characteristics of Patients With Oral Lichen Planus in a Cohort From Casablanca, Morocco

**DOI:** 10.7759/cureus.87015

**Published:** 2025-06-30

**Authors:** Soukaina Oujdad, Lamia Kissi, Mouna Hamza, Ihsane Ben Yahya

**Affiliations:** 1 Department of Oral Medicine and Oral Surgery, Faculté de Médecine Dentaire, Université Hassan II de Casablanca, Casablanca, MAR; 2 Department of Pediatric Dentistry and Epidemiology, Faculté de Médecine Dentaire, Université Hassan II de Casablanca, Casablanca, MAR

**Keywords:** clinical signs, lichen planus, malignant transformation, morocco, oral, prevalence

## Abstract

Background

Oral lichen planus (OLP) is a chronic inflammatory disorder that affects the oral mucosa and is more frequently observed in women. OLP presents in various forms, including reticular, papular, plaque-like, erosive, atrophic, and bullous. The etiology remains unclear, though immune dysregulation is thought to be a key factor. OLP has the potential to transform into oral squamous cell carcinoma (OSCC).

Materials and methods

We conducted a cross-sectional study at the Department of Oral Surgery and Pathology, University Hospital Ibn Rochd, Casablanca. Over a period of 46 months, patients diagnosed with OLP were followed. Data were collected using a standardized form that covered demographic, clinical, biological, and histological criteria, as well as follow-up information. Blood tests included complete blood count, glucose levels, and liver function tests. Biopsies were performed for diagnostic confirmation and follow-up, with direct immunofluorescence (DIF) used in complex cases. Patients with suspected cutaneous lichen planus (CLP) were referred to the dermatology department for evaluation. Treatment involved the removal of irritants, with corticosteroid therapy tailored to the severity of the lesion. Systemic corticosteroids were used for severe or treatment-resistant cases. Diabetic patients requiring systemic therapy were managed in collaboration with endocrinologists.

Results

Out of 9,841 consultations, 44 cases of OLP were identified, resulting in a prevalence rate of 4.5‰. The mean age of patients was 54.5 ± 13.5 years, with a predominance of women. Comorbidities included diabetes and hypertension in some cases. The reticular form was most common, followed by the bullous form. The buccal mucosa was the primary site of involvement. CLP co-occurred in 22.7% of cases. Malignant transformation was observed in one patient.

Discussion

The study, conducted at a single center with a focus on oral pathology, aligns with international findings, demonstrating a higher prevalence in women aged 50 to 60 years and a predominance of reticular forms with involvement of the buccal mucosa. A higher incidence of bullous lesions was observed compared to previous reports. Anti-HCV (hepatitis C virus) testing was systematically performed due to Morocco's moderate HCV prevalence. The association between OLP and diabetes may be influenced by the high diabetes prevalence in Morocco. Although treatment provided symptom relief, therapeutic options remain limited. The rate of malignant transformation was consistent with that reported in prior studies.

Conclusion

This study confirms a higher prevalence of OLP in women aged 50 to 60 years, with reticular forms and involvement of the buccal mucosa. Diabetes was the most commonly associated condition, and chronic HCV infection, while infrequent, may pose an increased risk. Regular monitoring is crucial due to the potential for malignant transformation, particularly in women and those with erosive or atrophic subtypes.

## Introduction

Oral lichen planus (OLP) is a chronic inflammatory mucocutaneous disorder driven by a T-cell-mediated autoimmune response, primarily targeting the basal keratinocytes of stratified squamous epithelium. It most commonly affects the oral mucosa but may also extend to other squamous-lined tissues such as the genital mucosa, skin, nails, and scalp. The pooled prevalence of OLP in the general population was estimated at 0.89%. The condition shows a marked female predilection and predominantly affects individuals aged 40 to 60 years [1].

Clinically, OLP presents with a spectrum of manifestations, from asymptomatic white keratotic plaques to painful erosions and ulcerations. The condition is classified into six distinct morphological subtypes: reticular, papular, plaque-like, erosive, atrophic, and bullous. The buccal, lingual, and gingival mucosae are the most commonly involved sites. Symptom severity varies widely, from incidental findings during routine examinations to severe burning pain, particularly in the erosive and atrophic forms [2].

The etiology of OLP remains elusive, though it is hypothesized to stem from a dysregulated immune response to specific antigens presented by innate immune cells and oral keratinocytes. This immune dysregulation leads to the upregulation of cytokines, chemokines, and adhesion molecules, thereby attracting T cells and mast cells to the affected tissues. The interaction among these immune cells results in keratinocyte apoptosis, disruption of the mucosal basement membrane, and the chronicity of the disease. CD8+ cytotoxic T cells, along with various T-helper cell subsets including Th9, Th17, and Tregs, are thought to play central roles in the pathogenesis of OLP [3].

The potential for OLP to undergo malignant transformation into oral squamous cell carcinoma (OSCC) is a matter of concern, leading the World Health Organization (WHO) to classify OLP as a disorder with malignant potential [4].

The primary aim of this study was to assess the prevalence of OLP in Casablanca, Morocco. Secondary objectives included the description of patients' demographic and clinical characteristics, the analysis of lesion distribution and clinical subtypes, the investigation of potential associations with systemic diseases, and the identification of any features suggestive of malignant transformation.

This article was previously presented as a poster at the 2021 MASCC/ISOO Annual Meeting on June 24, 2021.

## Materials and methods

A prospective study was conducted in the Department of Oral Surgery and Pathology at the Center for Dental Consultation and Treatment of Casablanca, University Hospital Ibn Rochd, involving patients diagnosed with OLP over a 46-month period, from December 1, 2015, to October 1, 2019.

Inclusion and exclusion criteria

Inclusion criteria encompassed patients diagnosed with OLP based on the clinical classification criteria established by the WHO [5]. Exclusion criteria comprised patients presenting with other oral potentially malignant disorders, mucosal lesions exhibiting malignant transformation, or those with insufficient clinical signs and a histological diagnosis inconsistent with OLP, such as pemphigus vulgaris.

Study procedure

Data collection was carried out using a standardized form comprising six sections: demographic criteria, general condition and medical history, clinical criteria, biological criteria, histological criteria, and follow-up.

All patients underwent a comprehensive evaluation, which included a complete blood count, a fasting blood glucose measurement, HCV (hepatitis C virus) screening, and liver enzyme analysis, specifically alanine aminotransferase (ALT) and aspartate aminotransferase (AST). Institutional Ethical Committee approval was obtained prior to the beginning of the survey (approval no. IRB-16/04). All procedures were conducted in accordance with the ethical standards of the institutional and/or national research committee, as well as the 1964 Declaration of Helsinki and its subsequent amendments.

A biopsy was performed whenever the clinical presentation of OLP was insufficient for diagnosis. A second biopsy was scheduled during follow-up if lesions exhibited changes suggestive of malignant transformation. Direct immunofluorescence (DIF) was requested when histological examination alone was inconclusive.

Patients with extraoral lesions evoking a cutaneous lichen planus (CLP) were referred to the dermatology department of the University Hospital of Ibn Rochd to confirm the diagnosis and ensure concurrent management.

A structured follow-up protocol was established for all patients, including periodic lesion examinations: weekly until clinical signs regressed, then every 15 days, and subsequently on a monthly basis. Photographs were taken at each visit to document disease progression. Histological examination was repeated whenever clinical changes suggested the presence of malignancy.

Management of confirmed cases involved eliminating local irritants such as ill-fitting prostheses, defective restorations, and carious lesions.

Corticosteroid therapy was adapted according to disease severity. Topical corticosteroids were preferred in cases of moderate inflammation, including prednisolone oral rinses and betamethasone or hydrocortisone tablets. Systemic corticosteroids were prescribed as a second-line treatment in cases of multiple lesions, erosive forms, or failure of topical therapy. The protocol included an induction dose of prednisolone at 1 mg/kg body weight per day for 10 days, followed by a gradual tapering regimen, reducing the total dose by 5 mg every 10 days. Treatment adjustments were made according to the progression of the lesions.

Diabetic patients requiring systemic corticosteroid therapy were referred to their endocrinologist before treatment initiation to ensure coordinated management.

Statistical data

IBM SPSS Statistics for Windows, Version 23 (Released 2015; IBM Corp., Armonk, New York), was used to analyze the collected information. Frequency analysis and percentage analysis were employed to describe the data using descriptive statistics, and continuous variables were described using means and standard deviations.

## Results

**Figure 1:**
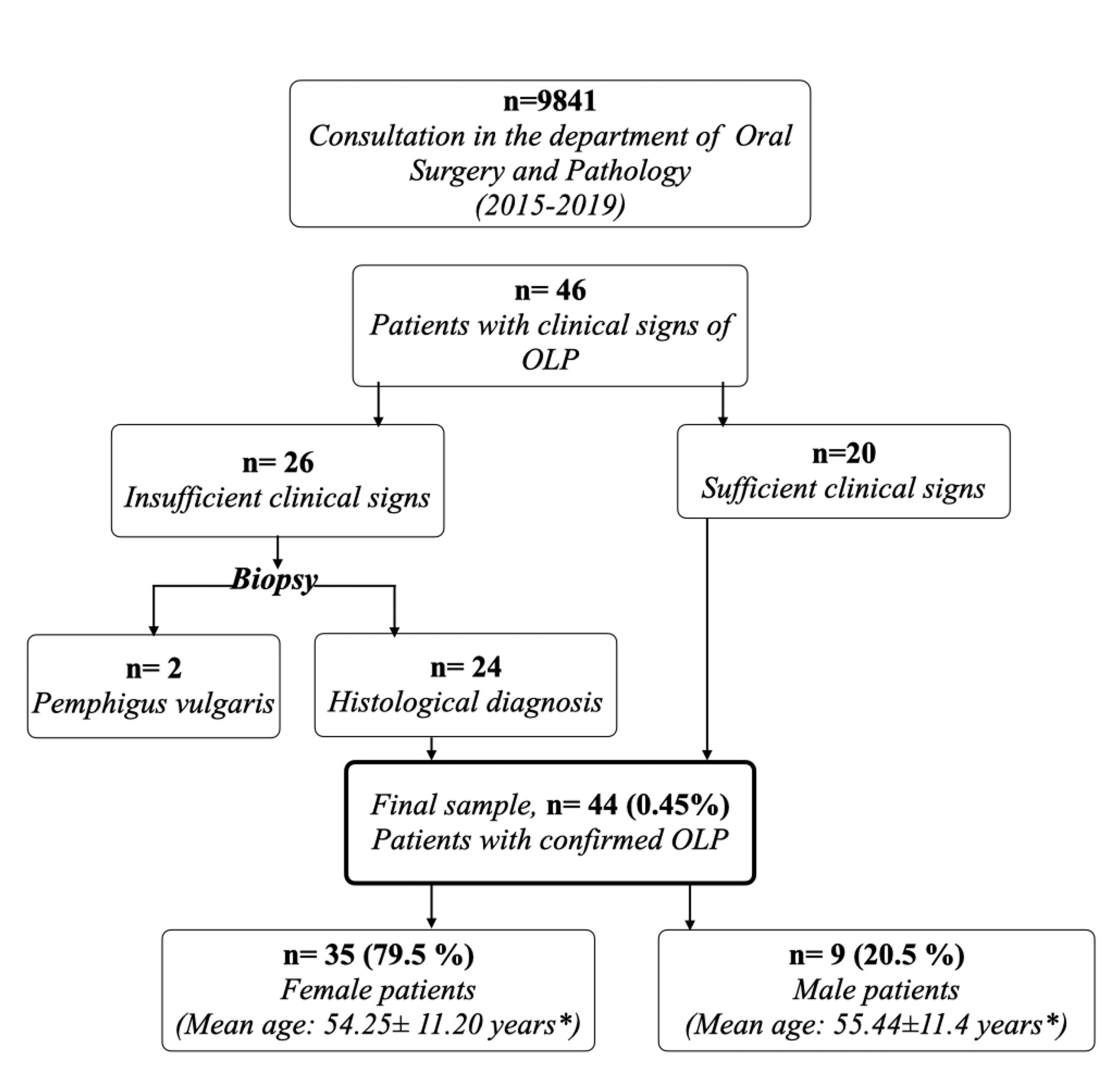
Demographical Distribution of Oral Lichen Planus (OLP) Among Patients Consulting in the Department of Oral Surgery and Pathology *: Mean age ± standard deviation

Out of a total of 9841 consultations, we identified 44 cases of OLP, resulting in an estimated prevalence of 0.45% (Figure 1). The majority of our patients were women, with a sex ratio of 1:3.87. The ages of the patients ranged from 24 to 81, with a mean age of 54.5±13.5 years. The most affected age group was between 51 and 60 years, accounting for 25% (n = 11) of women and 9.1% (n = 4) of men (Table 1).

**Table 1:** Age and Sex Distribution of Patients With Oral Lichen Planus *: Values are presented as n (%) **: Age is expressed as mean ± SD; ± denotes standard deviation (SD)

Age (Years)	Sex		Total
	Female	Male	
20-30	2 (4.5%)*	0 (0%)	2 (4.5%)
31-40	5 (11.4%)	1 (2.3%)	6 (13.6%)
41-50	5 (11.4%)	2 (4.5%)	7 (16%)
51-60	11 (25%)	4 (9.1%)	15 (34.1%)
61-70	8 (18.2%)	0 (0%)	8 (18.2%)
70≥	4 (9.1%)	2 (4.5%)	6 (13.6%)
Total	35 (79.5%)	9 (20.5%)	44 (100%)
Mean age ± standard deviation**	54.25±11.20	55.44±11.4	54.5±13.5

Among all patients, 27.3% had diabetes, 15.9% hypertension, 6.8% hypothyroidism, 4.5% hepatitis C, 2.3% a cardiac condition, and one patient (2.3%) was in remission from squamous cell carcinoma of the oral mucosa. The remaining individuals were not receiving treatment for any specific underlying health condition. A significant majority (88.6%) of the population were non-smokers, while instances of alcohol consumption (n = 1; 2.3%) and drug use (n = 1; 2.3%) were infrequent. None of the patients in our entire sample reported a family history of OLP (Table 2).

**Table 2:** Distribution of General Health Conditions, Lifestyle Factors, and Relevant Medical History Among Patients With OLP SCC: squamous cell carcinoma; OLP: oral lichen planus

Health Condition	Number of Patients	Percentage
No underlying condition	19	43.2%
Diabetes	12	27.3%
Hypertension	7	15.9%
Hypothyroidism	3	6.8%
Hepatitis C	1	2.3%
Cardiac condition	1	2.3%
Remission from SCC (oral mucosa)	1	2.3%
Alcohol consumption	1	2.3%
Drug use	1	2.3%
Non-smokers	39	88.6%
Family history of OLP	0	0%

During the initial consultation, most patients (95%) reported a range of symptoms, from mild discomfort to severe pain, with a prevailing sensation of burning. Only two patients (4.5%) had their OLP discovered incidentally (Table 3).

**Table 3:** Distribution of Functional Symptoms in Patients With Oral Lichen Planus

Functional Signs	Number of Patients	Percentage
Burning sensation	37	84%
Difficulty eating	30	68.2%
Xerostomia (dry mouth)	22	50%
Mucosal roughness	21	47.7%
Taste alteration	12	27.3%
Bleeding	8	18.2%

Assessment of oral hygiene status indicated that 26 patients (61.9%) exhibited good oral hygiene, 11 patients (26.2%) maintained a moderate level of hygiene, while five patients (11.9%) displayed inadequate oral hygiene. The oral hygiene status of the remaining two individuals could not be assessed, as they were fully edentulous and not wearing dentures at the time of clinical examination, pending stabilization of their OLP lesions.

**Figure 2:**
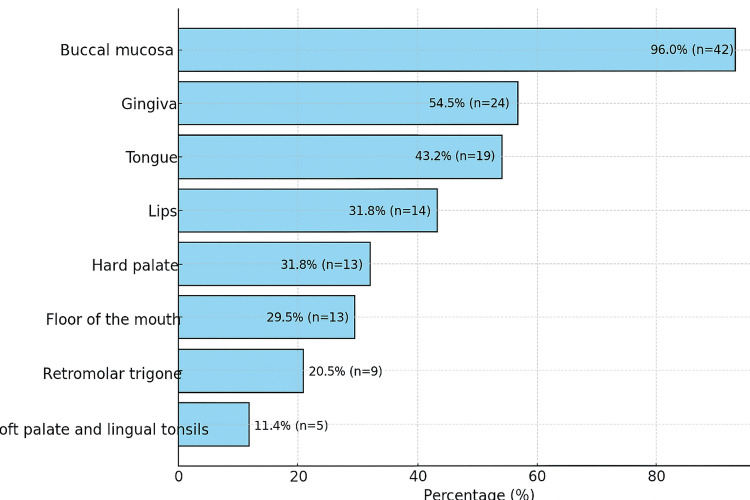
Distribution of Oral Lichen Planus According to Intraoral Localization

The majority of patients displayed multiple oral lesions of OLP, ranging from one to 10, with a prevailing occurrence on the buccal mucosa (n = 4%). The distribution of lesions based on their locations is outlined in Figure 2.

**Figure 3:**
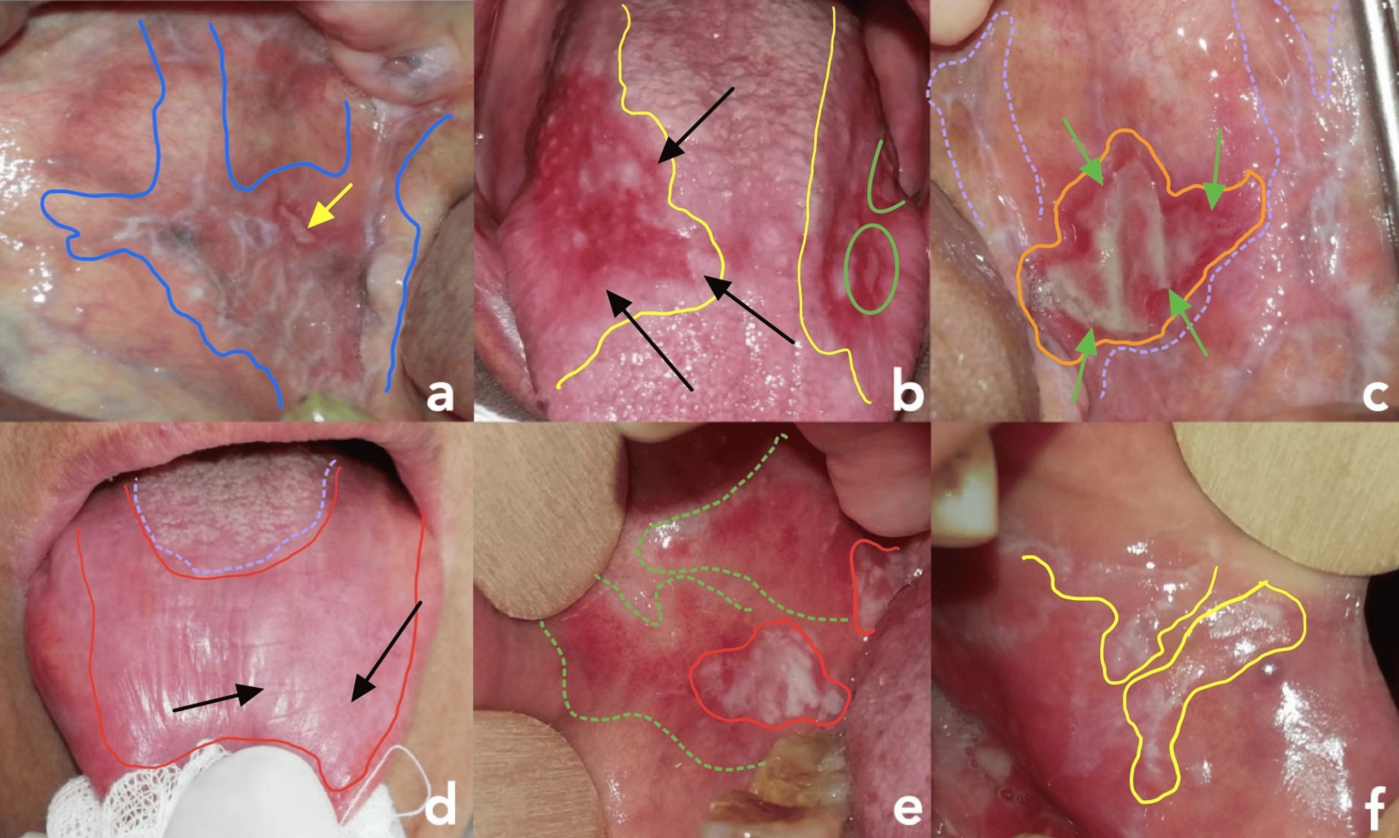
The Six Forms of OLP Identified in the Study (a) Reticular OLP: Reticular pattern involving the right buccal mucosa, characterized by interlacing white striae (*highlighted in blue*) and a centrally located ulcerative lesion (*yellow arrow*); (b) Erosive OLP: Two well-demarcated erosive lesions on the lateral borders of the tongue (highlighted in yellow), with extension to the dorsal surface showing depapillation erythema (black arrows) and focal ulceration (green circles); (c) Bullar OLP: Well-defined bullous lesion of oral lichen planus on the left buccal mucosa (highlighted in orange), bordered by a reddish inflammatory halo (green arrows). The lesion is superimposed on a dense reticular network of white striae (highlighted in purple), characteristic of the reticular subtype of OLP; (d) Atrophic OLP: Chronic atrophic lesion on the dorsal surface of the tongue, presenting as a large depapillated area (highlighted in red) contrasting with the posterior papillae-rich region (purple circle), with fine white striations overlying the dorsal mucosa (black arrows); (e) Plaque-like: Well-demarcated whitish plaque (highlighted in red) over an erythematous background on the buccal mucosa (green highlight); (f) Papular: Papular lesions forming a dense reticular network (yellow highlight) on the buccal mucosa near the labial commissure.

All six clinical forms of OLP were identified in our study. Most commonly, multiple clinical forms were noted in the same patient (Figure 3).

**Figure 4:**
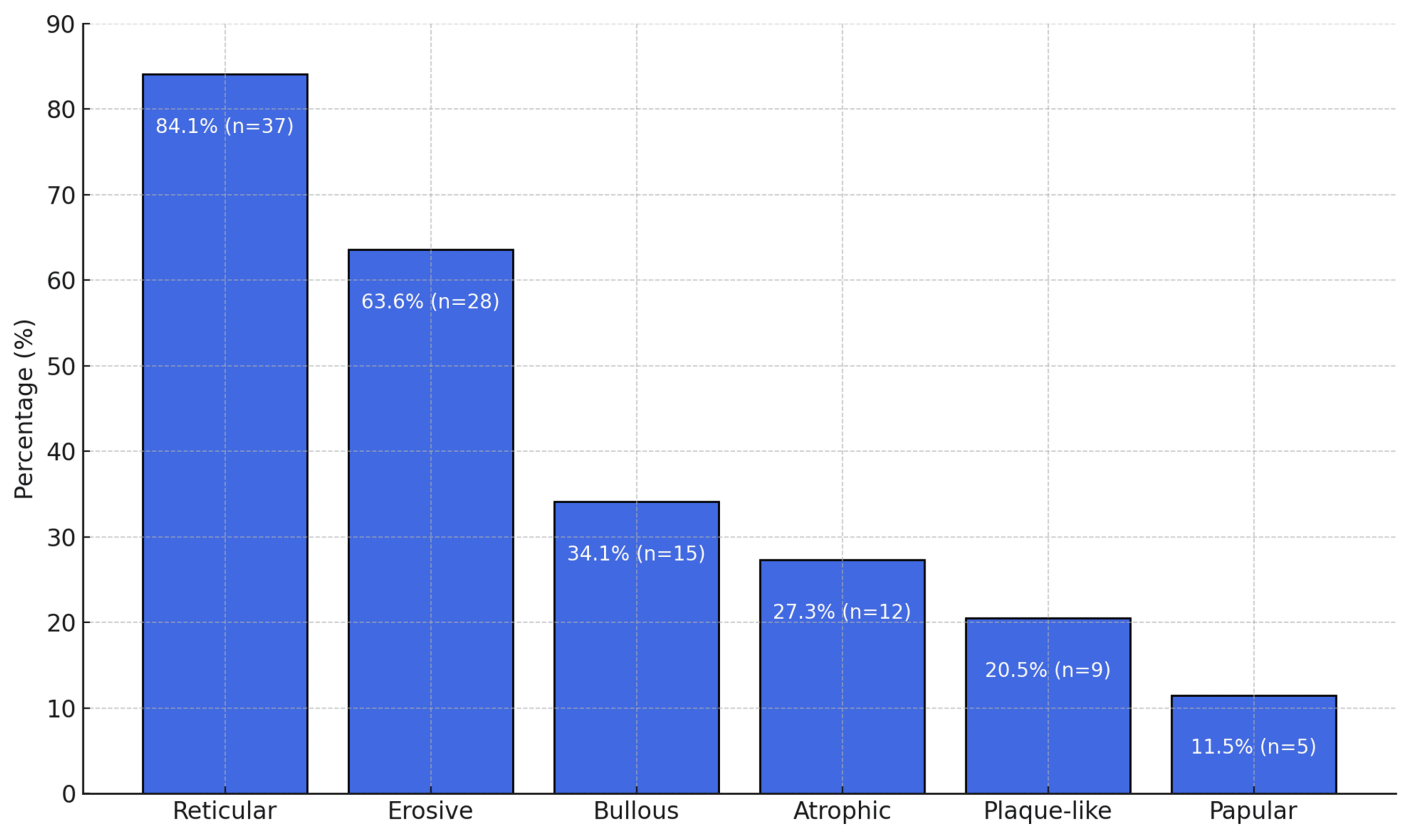
Distribution of Clinical Forms of Oral Lichen Planus

The reticular form was the most frequently observed, affecting nearly all patients, with a prevalence of 84.1%. The erosive form was present in 63.6% of cases. The distribution of the different forms is illustrated in Figure 4. CLP associated with OLP was observed in 10 patients (22.7%). These lesions were present on the chest, neck, forearms, and legs.

Systematic biological and serological tests were conducted on all patients. Of these, 16 (36.8%) patients had dysglycemia, and only one patient (2.3%) showed elevated AST/ALT levels.

A biopsy was performed on 24 patients (54.5%), none of whom exhibited signs of epithelial dysplasia. All patients were scheduled for periodic follow-up visits, with follow-up durations ranging from one to 30 months (mean: 6.68 ± 7.05 months). Clinical regression was reported in 82% of patients, whereas 16% experienced exacerbations and/or recurrences after treatment. Notably, one patient developed squamous cell carcinoma eight months after the initial consultation.

## Discussion

Our survey was a prospective cross-sectional study conducted over a 46-month period in the Department of Oral Medicine and Oral Surgery at the Faculty of Dental Medicine in Casablanca. The majority of reports in the literature were retrospective studies conducted over variable durations ranging from one to 40 years [5-10]. The prospective design of our study enabled us to address any deficiencies in patients' personal or clinical information, and it also provided us with the benefit of conducting effective clinical monitoring and documenting the various phases of disease progression.

Demographic data

Our population's traits exhibited many similarities to those reported in existing literature; however, we also identified some differences. The prevalence noted in our investigation, at 4.5 per thousand, was less than what has been reported in prior literature. This observation could be justified by the already established rarity of OLP, along with its lesser recognition and relatively limited familiarity among general practitioners.

As noted in other studies and confirmed in ours, there is a predominance of female patients, with a female-to-male sex ratio of M:F=1:3.9, which is consistent with many other studies [9,11]. Most studies agree that OLP occurs between the fifth and seventh decades of life, with a mean age of around 50 years. In our study, 34.1% of the subjects were aged between 50 and 60 years. The mean age (54.5 ± 13.5 years) was similar to that reported in Brazil (54.08 ± 13.14), the Czech Republic (55.2 ± 12.4), and Spain (56.35 ± 13.67) [9,12,13]. In our sample, no juvenile OLP was observed. Its occurrence in children is extremely rare, and very few cases have been reported in the literature [14].

Clinical presentation

In the majority of cases, patients exhibited involvement in multiple areas, with the buccal mucosa being the most commonly affected (96%), which aligns with findings from previous studies [8,11,12] reporting similar high rates of buccal mucosa involvement, ranging from 73% to 90%. Additional sites were affected, including the gingiva, tongue, labial mucosa, floor of the mouth, palate, and tonsils, with consistent results similar to those in other studies [5].

In our study, similar to what has been observed previously, the reticular and erosive forms were the most common. However, a notable difference was the relatively high rate of bullous OLP, which was documented at a rate of 34.1%. This percentage exceeded the rates reported in most studies, which typically range from 0% to 4% [15-17].

In our study, the association between OLP and CLP was found to be 22.7%, which is consistent with findings from Egypt (23.44%) [5] and Romania (25%) [18], less than those reported in Brazil (32.72%) [9] and higher than those reported in Iran (17%) [16]. Treatment for CLP typically depends on the location and severity of the lesions, guided by clinical expertise. Most lesions regress naturally within a few years, and unlike OLP, its cutaneous homologue is not considered premalignant. However, when mucosal sites such as the esophagus, larynx, or vulva are affected, a higher, though not clearly quantified, risk of malignant transformation has been reported [19].

OLP-systemic diseases

In our study, 27% of patients were diagnosed with diabetes, a proportion considerably higher than that reported in previous studies conducted in Croatia (5.2%), the Czech Republic (14.6%), and Romania (8.1%) [7,12,18]. This discrepancy may be attributed to the relatively high prevalence of diabetes in Morocco, estimated at 9.1% in 2021 [20]. Although several studies suggest a higher prevalence of diabetes among patients with OLP (and vice versa), the evidence remains inconsistent due to methodological heterogeneity and varying diagnostic criteria. Nevertheless, the potential link may be explained by shared immune-inflammatory mechanisms, particularly in type 2 diabetes. Given the clinical implications, including delayed healing and increased risk of complications, further well-designed studies are essential to clarify the nature and significance of this association [21,22].

Chronic HCV infection is a major global health issue and a leading cause of chronic hepatitis and liver cirrhosis. Globally, an estimated 50 to 58 million people are living with chronic HCV, with approximately one million new infections each year. In 2022, HCV was responsible for approximately 242,000 to 290,000 deaths worldwide [23].

The connection between OLP and HCV has been widely explored by various studies. Systematic reviews and meta-analyses consistently indicate a significant association between HCV infection and OLP, particularly in Mediterranean countries, the Middle East, and Asia. The varying prevalence of HCV in OLP patients across different geographic regions (e.g., 62% in northern Japan vs. 4% in northern France) suggests a link to regional HCV endemicity. This association suggests that HCV may contribute to OLP pathogenesis, possibly through an immunological response to HCV replication in the skin and oral mucosa [24,25].

According to the Oral Mucosa Study Group (GEMUB: Groupe d'Étude de la Muqueuse Buccale), routine HCV screening is not indicated for all patients with OLP. Instead, a personalized, risk-based approach is advocated, wherein serological testing is reserved for patients with individual risk factors or those presenting with erosive or treatment-resistant forms of OLP [26].

In Morocco, the estimated prevalence of chronic HCV infection in the general population ranges from 0.5% to 1.8%, placing the country in the low-to-intermediate category according to the WHO standards [27]. In light of these data, we support the implementation of targeted HCV screening in Moroccan patients with OLP, especially when clinical presentation (red forms of OLP) or patient history suggests an increased risk (drug intake).

Follow-up and treatment outcomes

The follow-up in our study ranged from one to 34 months. Most patients continued to attend their follow-up appointments throughout the entire observation period of the study.

The primary objective of treating OLP is to alleviate pain and reduce the chronic inflammation associated with the disease. Among the treated patients, 81.8% reported a regression of the clinical signs, while 15.9% experienced exacerbations and/or relapses after treatment. This worsening of symptoms was correlated with periods of emotional tension and/or significant anxiety and manifested as a recurrence of pain, discomfort, and visible inflammation that had previously been relieved by the initial treatment.

In our specific context, despite observing overall clinical improvement in most patients, it is important to underscore a major limitation in the Moroccan setting: the limited availability of key therapeutic agents commonly used in the management of OLP. These include topical and intralesional corticosteroids, as well as retinoids and oral calcineurin inhibitors specifically formulated for oral mucosal use. This therapeutic constraint has considerably narrowed our treatment arsenal.

Malignant transformation

The malignant transformation of OLP into SCC is the most concerning complication associated with OLP. In our study, only one case (2.27%) of malignant transformation was documented. This case involved a 62-year-old woman with rheumatoid arthritis who had no history of alcohol or tobacco use. The malignant transformation occurred on an erosive and bullous type of OLP, without any dysplasia noted in the initial biopsy.

OLP is classified among oral potentially malignant disorders (OPMDs) according to WHO-endorsed terminology and has a recognized potential for malignant transformation [4]. A systematic review by Fitzpatrick et al. reported transformation rates ranging from 0% to 3.5% across individual studies, with an overall rate of 1.09% for OLP [28]. Similarly, Giuliani et al. found an overall transformation rate of 1.40% for OLP. They emphasized the wide variability in reported rates, ranging from 0% to over 5%, and calculated an estimated annual transformation rate of 0.20% [29].

The malignant transformation of OLP most commonly affects the tongue, followed by the buccal mucosa. Furthermore, erosive or atrophic (red) subtypes and female gender are consistently reported as significant risk factors for this progression [28]. In our case, however, the transformation arose from erosive lesions located on the gingival mucosa of the edentulous ridge, a site less frequently reported in the literature [6].

In our study, we reported relatively low tobacco and alcohol consumption. Despite the lack of epidemiological evidence regarding its influence on the malignant transformation of OLP, its established carcinogenic potential should not be overlooked [28,29].

Limitations

Our study has its own limitations, including those inherent to all observational studies. Our study was conducted in a single center and was not comprehensive for the entire Moroccan population. Nevertheless, it is worth noting that the Consultation and Dental Treatment Center in Casablanca, part of the Faculty of Dental Medicine, has a significant concentration of oral pathology specialists in southern Morocco. The Department of Oral Medicine and Oral Pathology provides specialized consultations for oral mucosal pathologies on a daily basis, serving a large number of patients referred by general practitioners as well as other medical specialties.

## Conclusions

Based on our results, the epidemiological profile of patients in our sample demonstrates a clear female predominance, with the majority of patients falling within the 50 to 60-year age range. This observation directly supports our objective of describing the demographic features of the affected population. Clinically, the buccal mucosa emerged as the most frequently involved site, consistent with previous literature, while the reticular form was the most prevalent subtype. Notably, however, we identified a higher proportion of the bullous form than generally reported, which warrants further investigation. The primary reason for consultation was a burning sensation, emphasizing the functional impact of the disease. Approximately one-third of our cohort presented with an associated systemic condition, particularly diabetes, responding to our secondary objective of exploring associations with systemic diseases. Chronic HCV infection was low within our sample; however, based on published Moroccan data suggesting a possible link between OLP and HCV infection, we considered systematic HCV screening justified for high-risk patients.

In sum, our findings meet the stated objectives of assessing OLP prevalence, describing its demographic and clinical profile, and investigating associated systemic conditions. These findings also highlight the need for larger, multicenter studies to validate the results and optimize screening and management strategies for OLP in Morocco.
